# Lipid and Transcriptional Regulation in a Parkinson's Disease Mouse Model by Intranasal Vesicular and Hexosomal Plasmalogen‐Based Nanomedicines

**DOI:** 10.1002/adhm.202304588

**Published:** 2024-02-28

**Authors:** Yu Wu, Jieli Wang, Yuru Deng, Borislav Angelov, Takehiko Fujino, Md. Shamim Hossain, Angelina Angelova

**Affiliations:** ^1^ Université Paris‐Saclay Institut Galien Paris‐Saclay CNRS 17 Av. des Sciences Orsay 91190 France; ^2^ Wenzhou Institute University of Chinese Academy of Sciences No.1, Jinlian Road, Longwan District Wenzhou Zhejiang 325001 China; ^3^ Department of Structural Dynamics Extreme Light Infrastructure ERIC Dolni Brezany CZ‐25241 Czech Republic; ^4^ Institute of Rheological Functions of Food 2241‐1 Kubara, Hisayama‐cho Kasuya‐gun Fukuoka 811‐2501 Japan

**Keywords:** hexosomes, in vivo Parkinson's disease transgenic mouse model, liquid crystalline lipid nanoparticles, nanomedicine, RNA‐seq

## Abstract

Plasmalogens (vinyl‐ether phospholipids) are an emergent class of lipid drugs against various diseases involving neuro‐inflammation, oxidative stress, mitochondrial dysfunction, and altered lipid metabolism. They can activate neurotrophic and neuroprotective signaling pathways but low bioavailabilities limit their efficiency in curing neurodegeneration. Here, liquid crystalline lipid nanoparticles (LNPs) are created for the protection and non‐invasive intranasal delivery of purified scallop‐derived plasmalogens. The in vivo results with a transgenic mouse Parkinson's disease (PD) model (characterized by motor impairments and α‐synuclein deposition) demonstrate the crucial importance of LNP composition, which determines the self‐assembled nanostructure type. Vesicle and hexosome nanostructures (characterized by small‐angle X‐ray scattering) display different efficacy of the nanomedicine‐mediated recovery of motor function, lipid balance, and transcriptional regulation (e.g., reduced neuro‐inflammation and PD pathogenic gene expression). Intranasal vesicular and hexosomal plasmalogen‐based LNP treatment leads to improvement of the behavioral PD symptoms and downregulation of the *Il6*, *Il33*, and *Tnfa* genes. Moreover, RNA‐sequencing and lipidomic analyses establish a dramatic effect of hexosomal nanomedicines on PD amelioration, lipid metabolism, and the type and number of responsive transcripts that may be implicated in neuroregeneration.

## Graphical abstract

Liquid crystalline lipid nanoparticles derived from plasmalogen bioactive lipids (an emergent drug class for nanomedicine development against neurodegenerative disorders) are found to produce positive but distinct outcomes after 1‐month non‐invasive intranasal drug delivery in a transgenic mouse model of Parkinson's disease. RNA‐Seq and lipidomics identify key genes and lipid types responsive to plasmalogen‐based nanomedicine treatment.

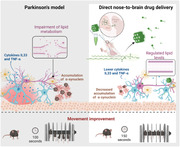

## 1 Introduction

Nanomedicine exploiting the self‐assembly of lipid molecules offers promises for non‐invasive treatment of neurodegenerative disorders (NDs) that may be caused by the accumulation of protein aggregates, misfolded proteins, oxidative stress, mitochondrial dysfunction, neuro‐inflammation, or lipid dysregulation.^[^ ^1^, ^2^, ^3^, ^4^, ^5^, ^6^, ^7^ ^]^ Phospholipids comprise bioactive agents, exerting a variety of therapeutic effects, as well as supramolecular‐structure‐forming materials, which interact with the biological barriers.^[^ ^8^, ^9^, ^10^ ^]^ Lipid nanoparticles (LNPs), a class of nanometric drug delivery systems, have been successfully used in the development of anti‐coronavirus vaccines and as reservoirs for the controlled delivery of various bioactive compounds.^[^ ^11^, ^12^, ^13^ ^]^ In fact, encapsulating therapeutic molecules within LNPs helps enhance their bioavailability and stability, reducing the drug dose, and avoiding severe side effects.^[^ ^14^, ^15^, ^16^, ^17^ ^]^ Using lipid‐based nanoparticles, including vesicles, cubosomes, hexosomes, spongosomes, nanoemulsions, nanostructured lipid carriers, and solid LNPs, offers nanomedicine‐mediated transport mechanisms and pathways for drugs reaching the targeted regions of the central nervous system.^[^ ^3^, ^4^, ^18^, ^19^, ^20^, ^21^ ^]^

Lipid replacement therapy (LRT) has emerged as a novel approach that aims to restore damaged cell membranes in pathological states.^[^ ^22^, ^23^, ^24^, ^25^, ^26^, ^27^, ^28^ ^]^ LRT may prove to be an alternative therapeutic option for the treatment of NDs, such as Parkinson's disease (PD), because many of these diseases are characterized by deficient lipid species.^[^ ^24^ ^]^ Studies have indicated that the concentrations of plasmalogens (Pls) decreased in both blood and brain of PD patients.^[^ ^29^, ^30^ ^]^ Such a deficiency has also been established in brains in animal models of early Alzheimer's disease (AD).^[^ ^31^ ^]^ Thus, Pls deficiency has been suggested to be a causative factor for PD, AD, and other NDs.^[^ ^17^, ^32^, ^33^ ^]^

Plasmalogens (Pls) constitute ≈30 mol% of total brain phospholipid content and about 70% of all glycerophospholipids in myelin. As a class of glycerophospholipids with a vinyl ether bond at the sn‐1 position of the glycerol backbone, Pls control the biomembrane structure, membrane protein activity, vesicular neurotransmitter release, and free radical scavenging.^[^ ^32^, ^34^ ^]^ Pls provide a storage depot for neuroprotective polyunsaturated fatty acids (PUFAs) such as docosahexaenoic acid (DHA).^[^ ^35^ ^]^ Treatment with plasmalogen precursor analogs has been shown to reduce dyskinesias in a PD monkey model.^[^ ^36^ ^]^ Moreover, oral administration of Pls has elevated blood plasmalogen levels and had a positive impact on the clinical symptoms of PD patients.^[^ ^37^ ^]^ Challenges that hinder the utilization of Pls as a drug include substantial oral metabolism, poor water solubility, limited oral bioavailability, and oxidation.^[^ ^30^ ^]^ The use of LNPs is suggested as a promising approach to improve Pls stability and enhance its delivery efficacy to the brain.^[^ ^38^ ^]^

The purpose of the present work is to utilize the advantages of designed internally nanostructured self‐assembled liquid crystalline lipid particles (LNPs) to enhance the efficacy of Pls lipid nano‐therapy via intranasal delivery to an animal model of NDs. Two types of LNP structures (vesicles and hexosomes) incorporating plasmalogens are investigated to gain insights into how the Pls‐based compositions and nanostructures may influence the impaired lipid metabolism and gene expression in the brain of transgenic PD mice after non‐invasive intranasal delivery. PD is a progressive neurodegenerative disorder characterized by motor abnormalities including bradykinesia (slowness of movement) and rigidity (stiffness).^[^ ^6^ ^]^ It is classified as an α‐synucleinopathy due to the accumulation of α‐synuclein protein,^[^ ^7^ ^]^ which may have diverse effects on the nervous system with implications in neuro‐inflammation and dysregulated lipid metabolism.^[^ ^22^, ^39^, ^40^, ^41^, ^42^ ^]^ The discovery of two missense mutations (*A53T* and *A30P*) in the α‐synuclein gene has provided valuable transgenic PD mouse models for deeper investigations of the pathological aspects and potential therapies of this disease.^[^ ^39^, ^42^ ^]^

There are limited reports about nanomedicine‐mediated regulation of gene and protein expression in the brain of transgenic PD mice. The chosen intranasal delivery route of Pls LNPs avoids the need for passage across the blood–brain barrier.^[^ ^43^, ^44^, ^45^ ^]^ The transport of Pls molecules is expected to proceed from the nasal cavity to the parenchyma of the brain along either olfactory or trigeminal nerves.^[^ ^46^ ^]^ We employed the RNA sequencing (RNA‐Seq) method to reveal the dynamic pool of RNAs present in hippocampal samples before and after treatment of the PD animals by Pls‐LNPs and determined the corresponding fold changes in gene transcripts after 1‐month intranasal delivery of LNPs. The regenerating capacity of Pls‐based vesicular and hexosomal LNPs was also evaluated by lipidomic analysis and in vitro assays.

## 2 Results and Discussion

### 2.1 Vesicular and Hexosomal Plasmalogen‐Based Nanomedicines

Nanoformulations of LNPs for intranasal delivery were prepared by dispersion of liquid crystalline nanoassemblies formed by purified plasmalogens (Pls) derived from natural scallops.^[^ ^38^ ^]^ The self‐assembled Pls/water mixtures were stabilized and dispersed by an amphiphilic surfactant (Pluronic) (see Table S1, Supporting Information, for lipid and amphiphilic compositions). A small quantity of a cationic lipid was incorporated into one of the nanoformulations with the purpose of enhancing the penetration of LNPs in nasal epithelium. The inclusion of a small quantity of the hydrophobic antioxidant vitamin E in the LNPs increased the mean packing parameter of the amphiphilic mixture and resulted in a structural transformation from a lamellar bilayer (AN1) to a nonlamellar topology (AN2) of the nanocarriers. Our experimental design considered the critical role of the topology and shape of LNPs in interacting with biological membrane barriers.^[^ ^19^, ^20^, ^21^ ^]^ In fact, the dynamics of these interactions could significantly impact the delivery efficiency of encapsulated drugs to living cells.

**Figure 1:**
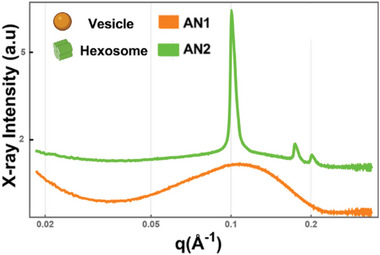
Synchrotron small‐angle X‐ray scattering (SAXS) patterns of plasmalogen‐based liquid crystalline dispersions of vesicular (AN1) and hexosome (AN2) types of lipid nanoparticle (LNP) (see Table S1, Supporting Information, for the compositions of AN1 and AN2 nanoformulations). Aqueous phase: 1 × 10^−2^ m phosphate buffer containing butylated hydroxytoluene (BHT). The temperature is 22 °C.

The performed synchrotron small‐angle X‐ray scattering (SAXS) investigation revealed two distinct structural organizations of plasmalogens‐based LNPs (**Figure** **1**). We identified stable LNPs of vesicular (referred to as AN1) and hexosome (referred to as AN2) types of structures. The positions of the Bragg peaks in the SAXS pattern of sample AN2 are indicative of the inverted hexagonal‐type periodic nanostructure, which diffracts the X‐rays. The *q*‐vector positions were used to determine the structural lattice parameter (lattice spacing *a*_HII_ = 6.7 nm) of the formed hexosome liquid crystalline nanoparticles (AN2).

In the case of sample AN1, the absence of Bragg diffraction peaks in the recorded scattering plot suggests that no periodic 3D assembled structures were formed upon hydration and dispersion of the Pls‐based mixture into nanoparticles. This observation indicates that scallop‐derived plasmalogens organized into vesicular structures (AN1) under the investigated dispersion conditions. The obtained vesicle and hexosome types of LNPs were investigated in a transgenic mouse model of PD with a focus on evaluating the efficacy after intranasal delivery and establishing the potential influence of nanostructure type on the therapeutic outcomes.

### 2.2 In Vitro Effect of Plasmalogen‐Based Vesicular and Hexosomal Lipid Nanoparticles on the Survival of Differentiated SH‐SY5Y Cells

**Figure 2:**
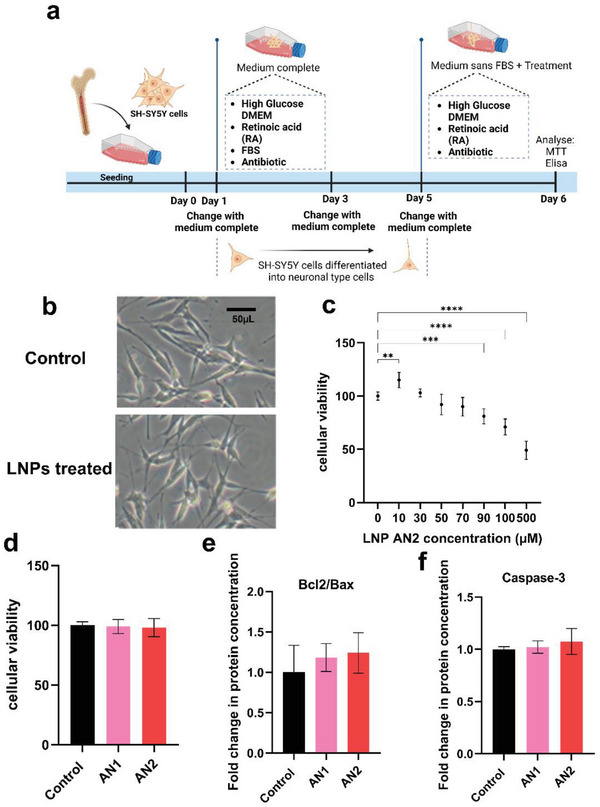
In vitro experiments showing the impact of plasmalogen‐based vesicular (AN1) and hexosomal (AN2) LNPs on cellular viability and apoptosis regulation in the differentiated SH‐SY5Y cells. a) A schematic diagram of experimental design, b) a comparison of cell morphology between control and treated groups, c) assessment of cellular viability after treatment with various AN2 concentrations for 24 h (*n* = 6), d) evaluation of cellular viability following 10 µm AN1 and AN2 treatments for 24 h (*n* = 6), e) measurement of Bcl2 and BAX expression via ELISA in SH‐SY5Y cells treated with 10 µm AN1 and AN2 for 24 h (*n* = 3), and f) determination of expressed caspase‐3 using ELISA under the same conditions (*n* = 3). All experiments were performed on SH‐SY5Y cells differentiated for 5 days with 10 µm RA. The control group received a culture medium without FBS. Statistical analyses included the Dunnett test for multiple comparisons and Student *t*‐test for two‐group comparisons, conducted using Prism software. All data are presented as mean ± SD. Significance levels are indicated as * *p* ≤ 0.05, ** *p* ≤ 0.01, and *** *p* ≤ 0.001.

To investigate the impact of Pls‐based vesicular (AN1) and hexosome (AN2) types of LNPs on differentiated SH‐SY5Y cells of a neuronal phenotype (a widely used in vitro model of neurodegeneration),^[^ ^38^ ^]^ we assessed the effects of LNPs on the cellular viability, as well as on the levels of a key enzyme (caspase‐3) and proteins (Bcl2 and Bax), involved in the cellular apoptotic pathways (**Figure** **2**). Following 5 days of cell differentiation with retinoic acid (RA), the performed morphological study showed that 10 µm LNP treatment for 24 h did not alter cellular morphology nor induced apoptosis‐related features (Figure 2b). Dose‐dependent assessments revealed that AN2 had no significant impact on cellular viability up to a concentration of 90 µm. A concentration of 10 µm enhanced the viability to 115% compared to control. Concentrations above 90 µm led to a notable decrease in cell viability, which was reduced to 49% at 500 µm. The comparison of AN1 and AN2 LNPs at 10 µm showed no different effects on cell viability after 24 h of incubation (Figure 2d).

The levels of Bcl2 and Bax were quantified by enzyme‐linked immunosorbent assay (ELISA) after Pls‐LNP treatment (Figure 2e). These proteins belong to the BCL‐2 family, which regulates apoptosis (programmed cell death) by controlling the release of cytochrome c from mitochondria and the permeabilization of mitochondrial membranes. Bcl‐2 is an anti‐apoptotic protein that inhibits cell death and promotes cell survival, while Bax is a pro‐apoptotic protein that promotes cell death. The Bcl2/Bax ratio, indicative of anti‐apoptosis regulation, increased after AN1 and AN2 treatments but without statistical significance (*p* = 0.6196 and *p* = 0.4661, respectively). Caspases are a family of protease enzymes that play a central role in the execution of apoptosis. No significant increase in caspase‐3 activity was observed following 10 µm AN1 and AN2 treatments for 24 h (Figure 2f). These findings collectively suggested the safe nature of the Pls‐based LNPs for apoptotic regulation and maintaining the cellular viability of differentiated SH‐SY5Y cells.

### 2.3 Plasmalogen‐Based Nanomedicines Attenuate Motor Impairment in a PD Mouse Model

**Figure 3:**
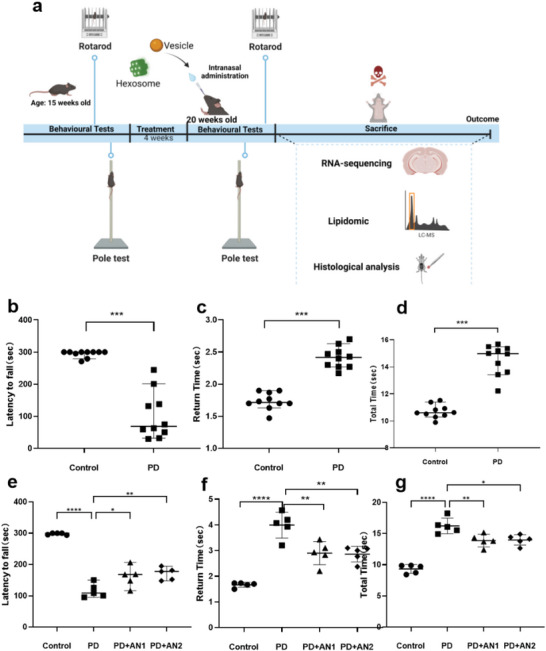
Behavioral tests of motor dysfunction in PD mice and improvement after intranasal plasmalogen‐LNP treatment. Transgenic PD mice displayed motor impairments at 15 weeks of age. The AN1 and AN2 LNPs attenuated this impairment after intranasal delivery for 4 weeks. a) Timeline of the in vivo experiments. b) Rotarod test with 15‐week‐old wild‐type mice and 15‐week‐old PD transgenic mouse (*n* = 10). c,d) Pole test with 15‐week‐old wild‐type mice and 15‐week‐old PD transgenic mouse (*n* = 10). e) Rotarod tests conducted on 20‐week‐old wild‐type mice (control), 20‐week‐old PD transgenic mouse (PD), and 20‐week‐old PD transgenic mice following a 4‐week treatment with AN1 and AN2 (PD+AN1, PD+AN2), respectively (*n* = 5). f,g) Pole tests were conducted on a 20‐week‐old wild‐type mouse (control), a 20‐week‐old PD transgenic mouse (PD), and a 20‐week‐old PD transgenic mouse following a 4‐week treatment with AN1 and AN2 (PD+AN1, PD+AN2), respectively (*n* = 5). Statistical analyses included the Dunnett test for multiple comparisons and Student *t*‐test for two‐group comparisons, conducted using Prism software. The data are expressed as the median ± 95% confidence interval. *p < 0.05, **p < 0.01, ***p < 0.001.

The performed in vivo study considered that the efficacy of nanomedicine treatment can depend on the choice of animal‐diseased model. Various PD models inducing α‐synuclein‐related neurodegeneration have been established, utilizing diverse promoters for the constitutive overexpression of familial‐PD‐associated mutant α‐synuclein forms (*A53T*, *A30P*, and *E46K*). These models manifest varying levels of neurodegeneration and replicate several clinical and biochemical PD features including oligomeric and fibrillar α‐synuclein formation. Our study employed transgenic mice carrying the *A53T* mutation site within the *Snca* gene. This in vivo model of PD was validated by the behavioral tests (**Figure** **3**). To assess the impairment of motor functions in transgenic PD mice, both rotarod and pole tests were conducted. The rotarod test was employed to evaluate the movement disorder in the PD model, where the latency to fall correlated positively with limb coordination and mouse grip strength. As expected, the wild‐type (WT) control group exhibited a significantly longer latency to fall compared to PD transgenic mice (Figure 3b). Motor function was further evaluated using the pole test (Figure 3c,d), revealing noticeable motor coordination deficits in the PD transgenic group, as evidenced by both return time and total time measurements. The average total time for the PD transgenic mouse group to turn at the top of the pole and descend was 14.53 s, contrasting with 10.7 s in the control group. The PD transgenic mice group displayed evident motion impairment. The validated PD transgenic mice model will be referred to as “PD mice” in the following context.

Rotarod and pole tests were also conducted to investigate the impact of Pls‐based vesicles (AN1) and hexosomes (AN2) on the motor function of treated animals. The results of the rotarod test revealed that PD mice were able to remain on the rod for an average of 116.1 s, whereas the AN1 and AN2 groups displayed significantly extended retention times of 161 and 170 s, respectively. Notably, both AN1 and AN2 groups exhibited significantly longer rod retention compared to PD mice (*p* < 0.05), as depicted in Figure 3e. Both AN1 and AN2 treated groups demonstrated reduced return times compared to the PD group. The AN1 and AN2 treated group exhibited improved motor coordination, taking 13.88 and 13.98 s, respectively, to ascend to the top of the pole and descend, as opposed to 16.2 s of the PD group. Intranasal administration of vesicles (AN1) and hexosomes (AN2) for 1 month established a significant restorative effect of the LNPs on PD‐induced movement impairment.

### 2.4 Regulation of Pathogenic PD Genes, Inflammatory Genes, and Lipid Metabolism Genes by Plasmalogen‐Based Vesicular and Hexosomal Nanomedicines in PD Mouse Model

**Figure 4:**
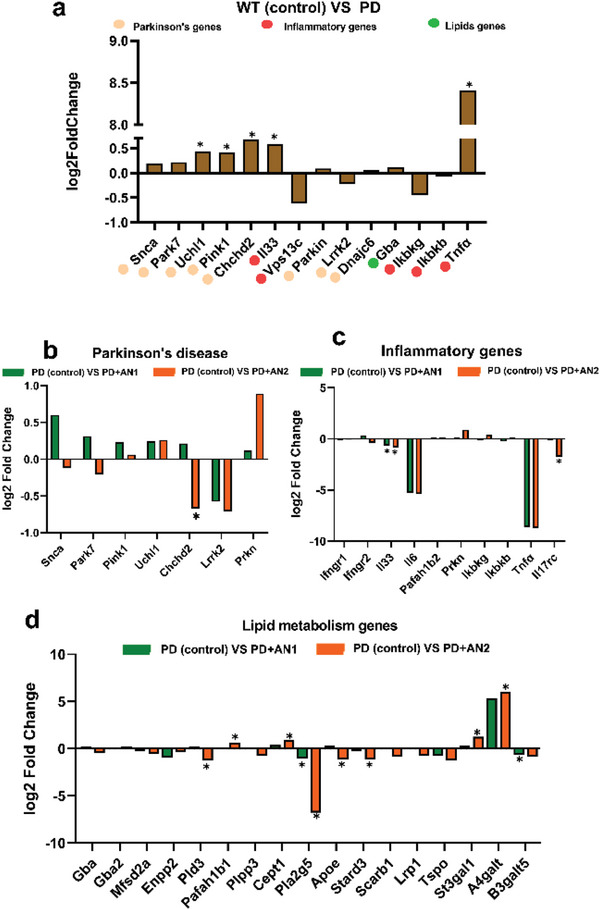
a) Parkinson's disease (PD) pathogenic gene expression in the hippocampus of the studied PD animal model. The fold change represents the gene expression in PD mice compared to wild‐type mice. b) Relative gene expression in PD mice was assessed after treatment with AN1 and AN2 LNPs. The PD group, serving as the control, received a vehicle (PBS) (*n* = 2). c) Inflammatory gene relative expression in the hippocampus of PD model after treatment with AN1 and AN2 LNPs. d) Lipid metabolism relative gene expression after treatments of PD mice with two types of LNPs: AN1 and AN2. DESeq2 analysis was performed. The data were plotted with GraphPad Prism 9. Data represented as the log2 fold change relative to control (*n* = 2). * *p* < 0.05.

We investigated whether the intranasal treatment of the PD model with Pls‐based LNPs may influence the dynamics of hippocampal gene expression considering that Pls are compounds characterized by anti‐apoptotic and anti‐inflammatory activities. The PD transgenic mice exhibited a log2‐fold increase of 0.19 in the *Snca* gene expression and 0.22 in the *Park7* gene expression as compared to WT mice (**Figure** **4**a). These are major pathogenic genes in PD, although the observed changes did not reach statistical significance (Padj = 0.297 and Padj = 0.15, as Figure 4a,b indicate). On the other hand, the expression of other genes related to PD, such as *Pink1*, *Chchd2*, and *Uchl1*, were significantly increased. The established changes in gene expression in PD mice versus WT validated the PD mouse model. The AN2 LNP treatment significantly decreased the expression of the PD‐related *Chchd2* gene, whereas AN1 did not significantly regulate genes related to PD pathology (Figure 4b). This difference may be due to the stronger penetration capacity of the AN2 LNPs, providing higher Pls concentration in the nasal mucosa and longer residence time upon transport to the PD mouse brain, as compared to the AN1 nanoformulation.

Considering that neuroinflammation also plays a key role in PD, the obtained results revealed an increase in the expression of genes related to inflammation (e.g., *Tnfα* and *Il33*) in the PD mouse model. Figure 4c shows that the LNP formulations reduced the *Il6* and *Il33* gene expression compared to the PD control. AN1 and AN2 LNP treatment downregulated *Tnfα* gene expression by 8.6‐fold and 8.7‐fold compared to PD mice, although statistical significance was not achieved (*p* = 0.07 and 0.06, respectively). Interestingly, LNPs AN2 reduced the expression of the *Il17rc* gene, which is involved in inflammatory responses, while LNPs AN1 had no significant impact on the expression of this gene.

Regarding the expression of genes related to lipid metabolism, vesicle (AN1) and hexosome (AN2) LNP‐treated mice exhibited different regulatory effects (Figure 4d). AN1 significantly downregulated the expression of *Pla2g5* and *B3galt5* genes. The AN2‐treated group significantly upregulated the expression of *Pafah1b1*, *Cept1*, *St3gal1*, and *A4galt*, while downregulating *Pld3*, *Pla2g5*, *Apoe*, and *Stard3* gene expression.

### 2.5 The Mitigating Effect of Plasmalogen on Cellular Proliferation in the Hippocampus of PD Mice

**Figure 5:**
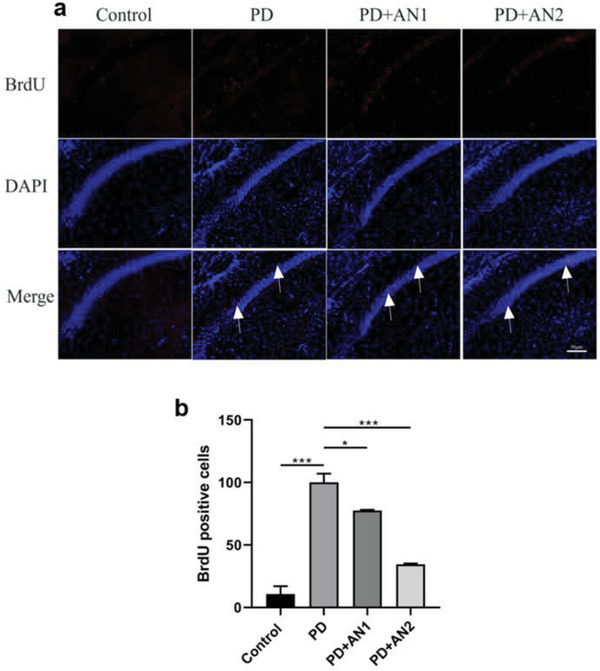
Visualization of proliferating cells in the mouse hippocampus by BrdU staining after plasmalogen‐based LNPs treatment of the PD model. a) BrdU incorporation (stained in green) and nuclei labeled with DAPI (stained in blue) in the regions of the hippocampus. b) Quantification of the total number of proliferating cells (BrdU positive). Statistical analyses included the Dunnett test for multiple comparisons and Student *t*‐test for two‐group comparisons, conducted using Prism software. All data presented as mean ± one standard deviation (*n* = 3). * *p* < 0.05. ** *p* < 0.005, and *** *p* ≤ 0.001.

In the brain hippocampus, microglia and astrocytes are the primary cell types with the potential to proliferate, particularly in response to injury, inflammation, or other pathological conditions. In order to assess cellular proliferation, we utilized a BrdU incorporation assay. The treatment with AN1 and AN2 LNPs had a significant influence on cell proliferation in PD mice. As depicted in **Figure** **5**, PD mice in the same region exhibited an average of 100 cells incorporating BrdU, whereas the groups treated with plasmalogen (AN1) and plasmalogen‐encapsulated nanoparticles (AN2) had 78 and 35 cells, respectively. The proliferation of cells in PD mice displayed a marked increase compared to the control group, which had 11 cells. The results suggested that the observed effect may be due to the proliferation of glia cells (microglia and astrocytes) rather than neuronal cells. Both AN1 and AN2 LNP treatments significantly alleviated the proliferation.

### 2.6 Lipid Remodeling by Plasmalogen‐Based Nanoformulations in PD Mice

The principal component analysis (PCA) in Figure S8a, Supporting Information, displays a 95% distribution interval in the form of an ellipse, with points that deviate significantly from the ellipse possibly indicating outliers. This outcome suggests that there are no noteworthy outliers within the four sample groups. Furthermore, PCA results reveal that the first principal component accounts for 50.2% of the variance, and the second principal component accounts for 13.4%. The meanings of the abbreviations of the lipid species shown in Figure S8b, Supporting Information, are as follows: BMP: bis(monoacylglycerol) phosphate, Cer: ceramides, FFA: free fatty acids, GM3: monosialogangliosides, LPA: lyso‐PA, LPC: lyso‐PC, LPE: lyso‐PE, LPI: lyso‐PI, LPS: lyso‐PS, PA: phosphatidic acids, PC: phosphatidylcholines, PC‐O: alkyl PC, PE: phosphatidylethanolamines, PE‐O: alkyl PE, PG: phosphatidylglycerols, PI: phosphatidylinositols, PS: phosphatidylserines, SL: sulfatides, SM: sphingomyelins.

In the volcano plot (Figure S8c,d, Supporting Information), both statistical significance (*p*‐value) and the extent of change (fold change) across all metabolites influenced by AN1 formulations compared to the PD model and control are presented. In comparison to the control group, the AN1‐treated group showed significant downregulation of LPC19:0, PC O‐34:3, and LPC16:0e, and upregulation of SL d18/1/22:1 h. When compared to the PD group, PG36:2 and PE38:4 species were downregulated. This suggests that the lipid profile of the AN1 LNPs‐treated group is closer to that of the PD group than to the control group.

On the contrary, in the AN2 LNPs‐treated group, when compared with the PD group, lipids such as FFA20:4, FFA20:3, FFA18:1, PE O‐40:5 (O‐18:1/22:4), PE O‐38:6 (O‐16:1/22:5), LPE18:1p, and PS 36:4 were significantly upregulated. Additionally, lipids, such as PE38:4 (18:0/20:4), PE38:3 (18:0/20:3), and PE34:2, were significantly downregulated. On the other hand, when compared with the control group, just SL d18:2/22:1 h and PC38:4 species were upregulated. These results suggest that the AN2 LNPs‐treated group exhibited a lipid profile more like the control group than the PD group. In summary, AN2 LNPs had a more substantial effect on lipid metabolism in the PD model compared to AN1 LNPs (Figure S9, Supporting Information).

### 2.7 Ether Lipid and Lysolipid Regulation by Plasmalogen‐Based LNP Intranasal Treatment in PD Mice

**Figure 6:**
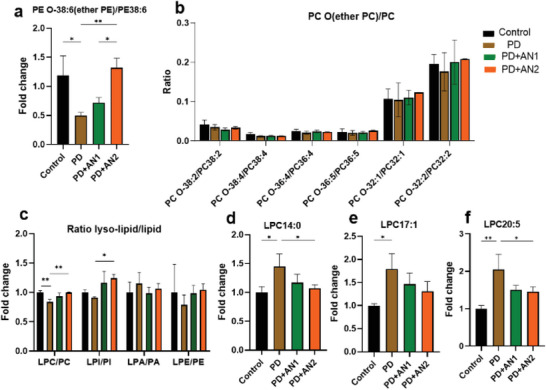
a) Fold change of the ratio of PE O‐38:6/ PE38:6. b) Determined ratio PC O/PC. c) Fold change of lysolipid levels. d) Fold change of LPC14:0. e) Fold change of LPC17:1. f) Fold change of LPC20:5. The Dunnett test was used for multiple comparisons, and the Student *t*‐test was employed for comparing two groups, both using Prism software. All data presented as mean ± one standard deviation (*n* = 3). * *p* ≤ 0.05, ** *p* ≤ 0.01, and *** *p* ≤ 0.001.

Ether phospholipids are a unique category of phospholipids, characterized by an ether bond instead of an ester link at the sn‐1 position of the glycerol backbone found in typical phospholipids. Ether lipids, including PA O, PC O, and PE O, provide essential cell functions within the body. They act as storage for specific substances and play roles in various diseases, particularly those affecting the brain. We found that in PD mice, the ratio of PE O 38:6 to PE 38:6 was lower compared to the control group. Following the administration of AN1 and AN2 LNP treatments to the mice, this ratio significantly increased when compared to the PD group. This suggests that these treatments could potentially recover lipid metabolism in PD mice to healthy levels (as shown in **Figure** **6**a). The ratio of PC O to PC did not change notably. Lysolipids are indispensable in providing structural support and facilitating cellular signaling processes, thus making significant contributions to the overall physiological functions of both individual cells and the entire organism. Our findings indicate that AN2 significantly increases lysophosphatidylinositol (LPI) compared to the PD group. PD group shows lower quantities of lysolipids in comparison to the control group, although these differences did not reach statistical significance (LPI P = 0.2, LPE P = 0.24, LPA P = 0.4). Notably, LPC14:0 was significantly elevated by PD, and its levels returned to those of the control group after AN2 treatment. LPC17:1 and LPC20:5 levels were significantly increased by PD, with AN2 reducing LPC20:5, while LPC17:1 remained unaffected by the treatment. This evidence suggests that the AN2 LNP treatment provokes positive responses in restoring lipid metabolism to its normal levels and provides an alternative avenue for addressing lipid metabolism concerns in PD. This emphasizes the importance of further studying their impact on the disease process.

In this study, we successfully prepared two plasmalogen‐based nanostructures, namely AN1 (vesicle) and AN2 (hexosome) LNPs, as confirmed by SAXS analysis (Figure 1). Upon incubation with neuronal SH‐SY5Y cells for 24 h at a concentration of 10 µm, we observed no signs of apoptosis or a decrease in cellular viability (Figure 2). Motion deficits represent a primary clinical feature and diagnostic criterion of PD. Prior research has indicated that plasmalogen administration via the intraperitoneal route can ameliorate motion impairment in mice with transient middle cerebral artery occlusion (tMCAO).^[^ ^34^ ^]^ Our findings demonstrate that intranasal delivery of plasmalogen‐based nanoparticles, both vesicles, and hexosomes, can attenuate motor deficits in transgenic PD mice, as measured by the pole test and rotarod test (Figure 3). These tests assess the muscle stiffness and rigidity commonly observed in PD, which hinder limb mobility and contribute to reduced motor function. These symptoms are primarily associated with the loss of dopamine‐producing neurons and subsequent dysfunction of the basal ganglia‐thalamo‐cortical pathway.^[^ ^47^ ^]^ The loss of motor ability results from complex dysfunction involving various factors including the accumulation of α‐synuclein (Figure S2, Supporting Information) and numerous PD‐related pathological genes (Figures S3–S6, Supporting Information). For instance, Fan et al. have reported motor defects in CHCHD2 p.Thr61Ile knock‐in mice.^[^ ^48^ ^]^ Our results indicate abnormal *Chchd2* gene expression in PD mice, characterized by a dramatic increase. Notably, this increase was significantly reduced following AN2 LNP treatment. However, in the AN1 LNP‐treated group, we did not observe significant regulation of the *Chchd2* gene but noticed an improvement in motor function. Although the precise correlation between the *Chchd2* gene and motor function remains unclear, increasing evidence suggests that the *Chchd2* gene plays a pivotal role in the motor function of PD.^[^ ^49^ ^]^ Apart from *Chchd2*, other significant genes associated with PD, including *Uchl1*,^[^ ^50^ ^]^ *Pink1*,^[^ ^51^ ^]^ *Vps13c*,^[^ ^52^ ^]^ and *Htra2*,^[^ ^53^ ^]^ exhibited abnormally increased expression in PD mice. Surprisingly, we did not observe any regulation in the expression of these PD‐related genes following treatment with a plasmalogen‐based LNP formulation.

Neuroinflammatory processes have been identified as key contributors to the pathogenesis and progression of PD in both human patients and animal models.^[^ ^54^, ^55^ ^]^ Neurodegeneration and neuronal loss in neurodegenerative diseases are primarily attributed to elevated levels of proinflammatory and neurotoxic mediators, such as *TNF‐α*, *IL‐6*, *IL‐33*, and others.^[^ ^56^ ^]^ In our study, we observed a significant increase in the expression of *Il33* and *Tnfa* in the PD mouse model. Treatment with either AN1 or AN2 LNPs led to a decrease in *Il33, Il6*, and *Tnfa* expression levels. Although the changes in *Il6* and *Tnfa* levels showed *p*‐values of 0.18, 0.19, 0.07, and 0.07 for AN1 and AN2, respectively, the observed variations in *Il6* and *Tnfa* levels may have clinical importance. Another hallmark of neuroinflammation is microglial activation, which is consistently observed in both PD patients and animal PD models.^[^ ^57^ ^]^ Activated microglia cells release a wide array of proinflammatory molecules, which can ultimately result in neuronal injury. Theodore et al. demonstrated that overexpression of α‐synuclein in mice indeed triggered microglial activation and the production of inflammatory cytokines.^[^ ^58^ ^]^ Our results confirmed an enhanced proliferation of microglia in the hippocampus in response to PD and a reduction in proliferation in AN1 and AN2 LNP treatment groups. Plasmalogens have been reported to reduce neuroinflammation and microglial activation through both oral and intraperitoneal administration.^[^ ^59^, ^60^ ^]^ However, the BrdU incorporation technique not only stains microglial cells but also newborn neurons in the hippocampus (neurogenesis), which complicates drawing a straight conclusion. Co‐staining with glial markers (Iba1 and GFAP) in future experiments may offer better insights into the context. In this study, we demonstrated the effectiveness of plasmalogens in reducing neuroinflammation in a PD mice model through intranasal delivery of LNPs. Furthermore, the demonstrated biodistribution in the mouse brain following a nasal delivery of nanoparticles suggests a potentially effective method of plasmalogen administration.^[^ ^61^ ^]^

Lipids play a pivotal role in several aspects of PD pathology. These aspects include their interactions with α‐synuclein, mutations in genes associated with lipid metabolism, modifications in lipid signaling pathways, and their involvement in oxidative stress and inflammation.^[^ ^22^ ^]^ Notably, mutations in the glucocerebrosidase (GBA) gene, responsible for encoding a lysosomal enzyme, represent significant and prevalent risk factors for PD.^[^ ^62^ ^]^ We observed an increase in the expression of the GBA gene in our transgenic PD mice, but it was not significant. Both AN1 and AN2 LNPs had no impact on this gene expression (Figure 4b,e). The heatmap results of the lipidomic analysis in all samples revealed alterations in the lipid profiles following AN1 and AN2 LNP treatments in PD mice (Figure S8b, Supporting Information). Additionally, the Volcano plot, which compared AN1‐treated PD mice to both PD and control groups, and AN2‐treated PD mice to PD and control groups (Figure S8c–f, Supporting Information), revealed that the AN2‐treated PD group exhibited a lipid profile resembling that of the wild‐type group. AN2 demonstrates the ability to restore lipid metabolism to normal levels. Lipid deficiency has drastic consequences for nervous tissue. Numerous studies involving PD patients, as well as animal and cellular PD models, have consistently observed alterations in lipid composition.^[^ ^63^, ^64^ ^]^ Zhao and colleagues using LC‐MS analysis identified 17 metabolites from blood plasma samples with significantly altered levels in PD patients.^[^ ^41^ ^]^ One hypothesis explaining the connection between PD and lipid metabolites is the potential role of PUFAs in promoting α‐Syn oligomerization within intact mesencephalic neuronal cells.^[^ ^64^ ^]^ Another hypothesis is that lipid metabolism disruption as one of the main pathogenic mechanisms in GBA‐associated PD.^[^ ^65^ ^]^ Figure S8a, Supporting Information, demonstrates that the ratio of ether PE 38:6/PE 38:6 was significantly reduced in the PD model. Both AN1 and AN2 LNPs were able to increase this ratio to levels similar to those of wild‐type mice. In the four groups (control, PD, two treated PD groups), the ratio of ether PC/PC showed no significant change. DHA is a vital component for supporting brain development and cognitive function and cannot be naturally produced within the brain. Nguyen et al. have provided evidence that highlights the significance of LPC in facilitating the transfer of DHA from plasma to the brain.^[^ ^66^ ^]^ Mulder et al. have reported a decrease in the lysophosphatidylcholine (LPC)/phosphatidylcholine (PC) ratio in cerebrospinal fluid in Alzheimer's disease.^[^ ^67^ ^]^ In our PD mouse model, Figure S8c, Supporting Information, shows that the LPC/PC ratio significantly decreased in the PD model, and AN2‐treated PD groups exhibited an increase, returning to control levels. The ratio of LPI/PI showed a slight reduction in the PD group without statistical significance (*p* = 0.7198), while AN2 also had an increasing effect on LPI/PI ratio in PD. Apart from the overall LPC/PC reduction in PD, three specific LPC species (LPC14:0, LPC17:1, LPC20:5) were significantly increased in PD. Following the AN2 treatment, their levels almost returned to control levels (Figure 6d‐f).

The influence of AN2 LNPs on lipid metabolism further corroborates with RNA expression changes, particularly the upregulation of *PAFAH1B1*,^[^ ^68^ ^]^ *CEPT1*,^[^ ^69^ ^]^ *ST3GAL1*,^[^ ^70^ ^]^ and *A4GALT* ^[^ ^71^ ^]^ genes. This genetic modulation may result in an alteration of lipid profile in the AN2‐treated PD group. *PLA2G5* gene encodes phospholipase A2 group V (PLA2‐V), an enzyme in the phospholipase A2 (PLA2) family. This enzyme produces bioactive lipids including lysophospholipids (LysoPLs), fatty acids (FAs), and eicosanoids.^[^ ^72^, ^73^ ^]^ Both AN1 and AN2 treatments significantly reduce *PLA2G5* gene expression. All the evidence indicates that plasmalogen encapsulated in hexosomes has a greater impact on PD model mice, with a higher capacity to restore PD pathologies to normal levels when compared to the control group. This outcome highlights the advantage of using lyotropic nonlamellar liquid crystalline (hexosomes) for intranasal brain delivery. This is attributed to their stability and mucoadhesive properties, which can enhance the retention time of nanoformulation in the nasal cavity.^[^ ^74^, ^75^ ^]^ Of note, we observed no toxic effects during the period of conducting the experiment (Figure S10, Supporting Information).

## 3 Conclusion

In summary, our findings confirm that in vivo plasmalogen administration through 1‐month intranasal delivery can alleviate motor impairments in PD mice. We observed dysfunction in lipid metabolism and neuroinflammation in the PD mouse model, while plasmalogen‐based nanoparticles were able to attenuate certain inflammation markers (IL‐6, IL‐33, and TNF‐α) and restore lipid metabolism to levels comparable to those seen in wild‐type healthy mice. Both types of studied LNPs (vesicles and hexosomes) were able to transport the bioactive plasmalogen lipid compound to the brain in a noninvasive way, through nasal delivery, by avoiding the BBB. The obtained results revealed that the non‐lamellar composition enhanced the penetration of Pls‐based LNPs in the nasal epithelium, respectively their residence time ensuring higher Pls concentration and drug bioavailability. Thus, the use of lyotropic non‐lamellar liquid crystalline nanodelivery systems can enhance the efficiency of plasmalogen treatment therapy on transgenic PD mice. This understanding is crucial for the development of plasmalogen‐based nanomedicines, or other bioactive therapeutic compounds encapsulated in LNPs for the potential treatment of PD.

## 4 Experimental Section

### Materials

The PUFA‐plasmalogen (vinyl ether) compound 1‐(1Z‐octadecenyl)‐2‐docosahexaenoyl‐sn‐glycero‐3 phosphoethanolamine (PL‐DHA‐PE) and DOTAP lipid were purchased from Avanti Polar Lipids, Inc. (Alabama) (99% purity). The composition of the purified scallop‐derived plasmalogen extract was characterized by the provider as a mixture of ethanolamine ether phospholipid (49.4%), choline ether phospholipid (24.9%), cholesterol (16%), and ceramide aminoethyl phosphonate (CAEP) (9.7%). This natural plasmalogen combination with 70% vinyl ether phospholipid content is referred to as scPL70 in the denotation of the lipid phase. Sterile phosphate‐buffered saline (PBS), Pluronic F127, RA, vitamin E (VitE), 2,6‐di‐tert‐butyl‐4‐methylphenol (BHT), and other reagents were purchased from Sigma‐Aldrich. Water of MilliQ quality (Millipore Corp., Molsheim, France) was used for the preparation of a phosphate buffer solution (NaH2PO4/Na2HPO4, 1 × 10^−2^ m, pH 7.4, p.a. grade, Merck).

### Nanoformulation of LNPs

LNPs were prepared by the method of hydration of a lyophilized thin lipid film followed by physical agitation in excess aqueous phase. The composition of the LNPs for the in vivo animal experiments comprised purified scallop‐derived plasmalogen scPL70, the lipid DOTAP, Vitamin E, and Pluronic F127. The mass lipid proportions yielding vesicular and hexosome types of LNPs are given in Table S1, Supporting Information. The preparation protocol included a dry film formation after lipid mixing and evaporation of the solvent under N2 gas, rehydrating the film with an excess buffer solution, and using ultra‐sonication to fragment the liquid crystalline assembly into nanoparticles. The buffer contained the antioxidant BHT, which ensured the oxidative stability of the aqueous formulations by suppressing the formation of lipid hydroperoxides. The phosphate/BHT buffer medium was purged by nitrogen gas (to eliminate the dissolved oxygen) and filtered through a 0.2 µm sterile filter (Millipore Corp.).

### Animals and Nanomedicine‐Mediated Drug Treatment

All animal experimental protocols were reviewed and approved by the Institutional Animal Care and Use Committee (IACUC), Wenzhou Institute, University of Chinese Academy of Sciences (No. WIUCAS21071223). 35 adult male WT (B6) and homozygous transgenic (TG, B6‐hSNCA A53T, Gempharmatech, China) mice were initially used for this study. The transgenic B6‐hSNCA A53T mice and non‐transgenic mice for the study were obtained from Gempharmatech (China). One group of WT animals was used for validating the PD model (*n* = 5). The initial design of the study thus included four experimental groups (*n*  =  5): WT group, PD group, AN1‐treatment group, and AN2‐treatment group. Successful induction of PD mouse after 15 weeks was validated by a rotational and pole behavior test, described below. PD mice were assigned randomly to three groups of five mice: 1) the control group receiving no treatment (WT), 2) intranasal drops of AN1 plasmalogen formulation, and 3) intranasal drops of AN2 plasmalogen formulation. Every mouse received a bilateral administration of intranasal drops (two times 10 µL volume) using a micropipette for 1 month of treatment applied once a day. Mice were quickly and firmly picked up by the scruff of the neck behind the ears with the thumb and index finger of the hand and held in a supine position with the head elevated. The mice were anesthetized before introducing formulation drops into the nasal cavity. Experiments were designed to minimize the number of animals used and their suffering.

### Rotarod Test

The experiment was conducted over three consecutive working days, with the first 2 days designated for training and the third for the official test. On the initial day, the shaft rotated (ROTA ROD, 47 650 UGO BASILE) at a constant speed of 20 rpm, with continuous rodent treadmill training lasting 300 s (mice were replaced on the shaft if they fell off, and each mouse had to complete the 300‐s training). On the second day, the shaft continued to rotate at 20 rpm, with continuous rodent treadmill training extended to 600 s. Similarly, mice were replaced upon falling, and each had to complete the 600‐s training. On the third day, again at 20 rpm, the shaft recorded the time from the onset of the mouse's movement until it fell off, with each mouse undergoing this measurement three times. The maximum test duration was set at 300 s; if a mouse reached this limit during any test, further measurements were unnecessary, and the experiment concluded. Post‐experiment, the acquired data was analyzed and organized.

### Pole Test

The experiment was conducted over three consecutive working days: 2 days for training and 1 day for the formal test. On the first training day, mice were positioned head‐down at the top of a 60 cm climbing pole and allowed to descend using both feet. This process was repeated three times for each mouse. The second day focused on training the mice to turn their heads up while positioned head‐up on the top of the climbing pole and then descend to the ground with all four feet. Again, each mouse underwent three training repetitions. On the third day, the formal test took place. Mice were positioned head‐up at the top of the climbing pole, and the time it took for them to turn their heads down (Return Time) and the overall time for the entire process (Total Time) were recorded. These measurements served as an assessment of motor coordination in mice.

### RNA Sequencing

Total RNA was isolated using a TRIzol total RNA extraction kit (TIANGEN,Cat.No.DP424), which yielded >2 µg of total RNA per sample. RNA quality was examined by 0.8% agarose gel electrophoresis and spectrophotometry. High‐quality RNA with a 260/280 absorbance ratio of 1.8–2.2 was used for library construction and sequencing. Illumina library construction was performed according to the manufacturer's instructions (Illumina, USA). Oligo‐dT primers were used to transverse mRNA to obtain cDNA (APExBIO, Cat. No. K1159). Amplified cDNA for the synthesis of the second chain of cDNA. Purified cDNA products by AMPure XP system (Beckman Coulter, Beverly, USA). After library construction, library fragments were enriched by PCR amplification and selected according to a fragment size of 350–550 bp. The library was quality‐assessed using an Agilent 2100 Bioanalyzer (Agilent, USA). The library was sequenced using the Illumina NovaSeq 6000 sequencing platform (Paired end150) to generate raw reads. Raw paired‐end fastq reads were filtered by TrimGaloreto discard the adapters and low‐quality bases via calling the Cutadapt too.^[^ ^76^ ^]^ The clean reads obtained were then aligned to the mm10/hg19 mouse genome using HISAT2,^[^ ^77^ ^]^ followed by reference genome‐guided transcriptome assembly and gene expression quantification using StringTie.^[^ ^78^ ^]^ Differentially expressed genes (DEGs) were identified by DEseq2(for the sample with replications) ^[^ ^79^ ^]^ or edgeR (for the sample with no replication) ^[^ ^80^ ^]^ with a cut‐off value of log2|fold‐change|>1 and *p*‐adjust <0.05. The clusterProfiler ^[^ ^81^ ^]^ was used to perform functional enrichment analysis for the annotated significant DEGs, the potential genes in identified modules based on gene ontology (GO) and KEGG pathway categories. Terms with *p*‐value < 0.05 were considered significant. Gene set enrichment analysis (GSEA) was performed by the function in package clusterProfiler with a gene list sorted by log2 fold‐change.

### BrdU Staining

To prepare a working concentration of 5 mg mL^−1^, 1 mg of BrdU was diluted in 200 µL sterile PBS. Mice were injected intraperitoneally with 200 µL (1 mg) of BrdU solution. The incorporation of BrdU in the small intestine was detectable within 1 h after injection, and BrdU could be detected in most tissues after 24 h. The mouse was anesthetized, and its chest was opened to expose the heart. The right atrial appendage was cut open. Initially, 0.9% normal saline was infused through the left ventricle until clear liquid flowed out from the liver. Subsequently, perfusion fixation was completed using 4% PFA. The fixation was considered complete when the mouse convulsed, and the entire body became stiff. The complete brain tissue of the mouse was then extracted and post‐fixed in 4% PFA for 24 h. After post‐fixation, the brain tissue was immersed in a sucrose gradient solution, followed by direct preparation of frozen sections once the tissue was precipitated. Serial frozen sections with a thickness of 6 µm were made from 3.5–5 mm behind bregma and attached to glass slides. To rupture the membranes, the tissue slices were air‐dried at room temperature for 15 min, followed by the addition of 0.4% Triton and incubation at room temperature for 20 min. For restoration, the tissue sections were washed with PBS three times for 5 min each, and microwave heat repair was performed using 0.01 mmol L^−1^ citric acid. The sections were subjected to high heat for 5 min, low heat for 20 min, and allowed to cool naturally for ≈1 h. To denature the DNA, the tissue sections were washed with PBS three times for 5 min each and then treated with 2 m HCl at 4 °C for 30 min. Refolding was achieved by washing the tissue sections with PBS three times for 5 min each and then adding 0.01 m Tris‐HCl at room temperature for 10 min. For blocking, the tissue sections were washed with PBS three times for 5 min each and then blocked with goat serum for 2 h. Incubation with the primary antibody involved discarding the excess goat serum from the tissue slices without washing and adding the BrdU antibody, followed by overnight incubation at 4 °C. Incubation with the secondary antibody required re‐warming the tissue slices at 37 °C for 1 h, followed by adding the secondary antibody and incubating at 37 °C for 1 h. After incubation, the tissue sections were washed with PBS three times for 5 min each, and the nuclei were counterstained with DAPI for 5–8 min. Finally, the tissue sections were washed with PBS three times for 5 min each and sealed with glycerol. Fluorescence microscope images were captured for analysis.

### Tissue Examination

Tissue samples were examined, and the amount of tissue fixative used was ≈4 to 10 times the volume of the tissue. If the tissue was too thick, it was cut into several small pieces to ensure better fixation. Complete fixation of the mouse was achieved after 24 h of routine tissue fixation, indicated by grayish color, hardened texture, and bloodless cut surface. Dehydration of the tissue was performed only after complete fixation. In cases of inadequate fixation, the tissue was replaced with fresh fixative and further fixed.

### Tissue Trimming and Embedding

The refined tissue was placed cut‐side down into an embedding box, and the corresponding tissue number was marked. The trimmed tissue was then dehydrated. For embedding, the tissue box was transferred to the paraffin liquid cylinder clip of the paraffin embedding machine. The tissue block was taken, cut side down, and laid flat in the embedding frame. Tweezers were used to gently flatten the tissue block, followed by condensing on a cold stage. Once the wax block solidified, it was carefully removed from the embedding frame and stored below 30 °C.

### Tissue Sectioning

The embedded tissue was sliced using a paraffin microtome, with the section thickness generally set at 5 microns. Consistency in the starting position of tissue slices from the same type of tissue was ensured.

### Preparation of Tissue Slides

The sliced sections were unfolded in a warm water bath (water temperature 42–45 °C). Clean glass slides were used to pick up the sections and ensure smooth adhesion to the slide. The slides were placed in an oven at 37 °C for 24–48 h or at 60 °C for 1–2 h to firmly attach the sections to the glass slide, preventing detachment during the staining process. Ordinary slides were used for H&E or other histochemical staining, while anti‐off slides were used for IHC or immunofluorescence staining. 1–3 cut surfaces were taken from each slide, ensuring flatness and the absence of air bubbles. The section numbers corresponded to the wax blocks.

### Staining Procedure

The dried slides were subjected to a 30‐min dewaxing process by immersion in a staining jar filled with xylene. Subsequently, the slices were sequentially immersed in ethanol of increasing concentration: 100% – 95% – 80% – 75% – 50% – 30%, for 5 min each, followed by immersion in pure water. Harris hematoxylin solution was applied for 2–4 min for staining. The slices were rinsed under running water for 5 min. Differentiation was carried out by briefly immersing the slices in 1% hydrochloric acid and 70% alcohol to remove excess hematoxylin. A 10‐min wash under running water followed. Eosin solution was applied for ≈30–40 s for staining (if eosin staining was too intense, color separation was performed using 75% ethanol). Dehydration and transparency were achieved by immersing the slices in the following reagent staining vats: 95% ethanol for 1 min, 100% ethanol I for 2 min, 100% ethanol II for 3 min, xylene I for 5 min, and xylene II for 5 min. The slides were mounted with neutral gum, air‐dried for ≈10 min in a fume hood, and observed and captured using a microscope.

### Immunohistochemistry

The experimental protocol included the following steps. Tissue sectioning: Embedded the tissue and sliced it using a paraffin microtome (section thickness: 3–5 microns). Ensured consistent starting positions for the tissue slices. Tissue scooping: Unfolded the slices in a warm water bath (42–45 °C) and scooped them onto clean glass slides for smooth adhesion. Incubated the slides at 37 °C for 24–48 h or at 60 °C for 1–2 h to firmly attach the sections and prevent detachment during staining. H&E staining used regular slides, while IHC staining used anti‐off slides. Took 1–3 cut surfaces from each slice, ensuring flatness and no air bubbles. Section numbers corresponded to wax blocks. Dewaxing/hydration: Incubated sections in xylene for three times, 5 min each. Incubated in 100% ethanol twice for 10 min each, followed by 95% ethanol twice for 10 min each. Washed the sections twice with dH2O for 5 min each. Citrate retrieval: Immerseed sections in 1× citrate retrieval solution and microwaved until boiling; continued at sub‐boiling temperature for 10 min (95–98 °C). Cooled sections on the benchtop for 30 min. Washed sections with dH2O three times for 5 min each. Immunohistochemistry: Incubated sections in 3% aqueous hydrogen peroxide solution for 10 min. Washed sections twice with dH2O for 5 min each. Washed sections with wash buffer for 5 min. Blocked sections with blocking solution for 1 h at room temperature. Removed the blocking solution and added diluted primary antibody. Incubated overnight at 4 °C. Washed sections three times with wash buffer for 5 min each. Detection and staining: Applied SignalStain Boost Detection Reagent (HRP, Mouse #8125) and incubated for 30 min at room temperature. Washed sections three times with a wash buffer for 5 min each. Applied SignalStain DAB Chromogen and Substrate for proper staining intensity. Washed sections twice with dH2O for 5 min each. Dehydration and mounting: Incubated sections in 95% ethanol twice for 10 s each, followed by 100% ethanol twice for 10 s each. Incubated in xylene twice for 10 s each. Mounted slides with coverslips and mounting medium.

### Lipidomic Analysis

Lipids were extracted from 20 µL of plasma using a modified version of Bligh and Dyer's method as described previously.^[^ ^6^ ^]^ Briefly, 750 µL of chloroform:methanol:MilliQ H2O (3:6:1) (v/v/v) mixture was added to plasma samples and incubated at 1500 rpm for 30 min at 4 °C. At the end of the incubation, 350 µL of deionized water and 250 µL of chloroform were added to induce phase separation. The samples were then centrifuged and the lower organic phase containing lipids was extracted into a clean tube. Lipid extraction was repeated once by adding 450 µL of chloroform to the remaining aqueous phase, and the lipid extracts were pooled into a single tube and dried in the SpeedVac under OH mode. Samples were stored at −80 °C until further analysis. Lipidomic analyses were conducted at LipidALL Technologies using a Shimadzu Nexera 20‐AD coupled with Sciex QTRAP 6500 PLUS as reported previously.^[^ ^82^ ^]^ Separation of individual lipid classes of polar lipids by normal phase (NP)‐HPLC was carried out using a TUP‐HB silica column (i.d. 150 × 2.1 mm, 3 µm) with the following conditions: mobile phase A (chloroform:methanol:ammonium hydroxide, 89.5:10:0.5) and mobile phase B (chloroform:methanol:ammonium hydroxide:water, 55:39:0.5:5.5). MRM transitions were set up for comparative analysis of various polar lipids. Individual lipid species were quantified by referencing to spiked internal standards. d9‐PC32:0(16:0/16:0), d9‐PC36:1p(18:0p/18:1), d7‐PE33:1(15:0/18:1), d9‐PE36:1p(18:0p/18:1), d31‐PS(d31‐16:0/18:1), d7‐PA33:1(15:0/18:1), d7‐PG33:1(15:0/18:1), d7‐PI33:1(15:0/18:1), C17‐SL, C14‐BMP, d9‐SM d18:1/18:1, d7‐LPC18:1, d7‐LPE18:1, C17‐LPI, C17‐LPA, and C17‐LPS were obtained from Avanti Polar Lipids. GM3‐d18:1/18:0‐d3 was purchased from Matreya LLC. Free fatty acids were quantitated using d31‐16:0 (Sigma‐Aldrich) and d8‐20:4 (Cayman Chemicals).

### Statistical Analysis

Analytical data were expressed as mean ± standard error of the mean or as the median ± 95% confidence interval. Differences between groups were determined using one‐way analysis of variance (ANOVA) followed by the Dunnett‐*t* test for multiple comparisons. Student *t*‐test for two‐group comparisons. *p*‐values: *p < 0.05, **p < 0.01, and *** *p* ≤ 0.001.

## Conflict of Interest

The authors declare no conflict of interest.

## Supporting information

Supporting Information

## Data Availability

The data that support the findings of this study are available in the supplementary material of this article.
