# Supplementary material for: Lipid and Transcriptional Regulation in a Parkinson's Disease Mouse Model by Intranasal Vesicular and Hexosomal Plasmalogen‐Based Nanomedicines
Source: Adv Healthc Mater. 2024 Feb 28;13(14):2304588. doi: 10.1002/adhm.202304588 (PMC11468381; doi:10.1002/adhm.202304588)
Supplement: Supplementary file 1 — Supporting Information [file ADHM-13-2304588-s001.pdf]

ADVANCED HEALTHCARE MATERIALS

# Supporting Information

for *Adv. Healthcare Mater.*, DOI 10.1002/adhm.202304588

Lipid and Transcriptional Regulation in a Parkinson's Disease Mouse Model by Intranasal Vesicular and Hexosomal Plasmalogen-Based Nanomedicines

*Yu Wu, Jieli Wang, Yuru Deng*, Borislav Angelov, Takehiko Fujino, Md. Shamim Hossain and Angelina Angelova**

Supporting Information
©Wiley-VCH 2024
69451 Weinheim, Germany

# Lipid and Transcriptional Regulation in a Parkinson's Disease Mouse Model by Intranasal Vesicular and Hexosomal Plasmalogen-based Nanomedicines

Yu Wu[1], Jieli Wang[2], Yuru Deng[2,*], Borislav Angelov[3], Takehiko Fujino[4],
Md. Shamim Hossain[4] and Angelina Angelova[1,*]

**Abstract:** Plasmalogens (vinyl-ether phospholipids) are an emergent class of lipid drugs against various diseases involving neuro-inflammation, oxidative stress, mitochondrial dysfunction, and altered lipid metabolism. They can activate neurotrophic and neuroprotective signaling pathways but low bioavailabilities limit their efficiency in curing neurodegeneration. Here we created liquid crystalline lipid nanoparticles (LNPs) for protection and non-invasive intranasal delivery of purified scallop-derived plasmalogens. Our *in vivo* results with a transgenic mouse Parkinson's disease (PD) model (characterized by motor impairments and α-synuclein deposition) demonstrated the crucial importance of LNP composition, which determines the self-assembled nanostructure type. Vesicle and hexosome nanostructures (characterized by SAXS) displayed different efficacy of the nanomedicine-mediated recovery of motor function, lipid balance, and transcriptional regulation (e.g., reduced neuro-inflammation and PD pathogenic gene expression). Intranasal vesicular and hexosomal plasmalogen-based LNP treatment led to improvement of the behavioral PD symptoms and downregulation of the *Il6*, *Il33,* and *Tnfa* genes. Moreover, RNA-sequencing and lipidomic analyses established a dramatic effect of hexosomal nanomedicines on PD amelioration, lipid metabolism, and the type and number of responsive transcripts that may be implicated in neuroregeneration.

DOI:10.1002/adhm.202304588

Table of Contents

Abbreviations list .......... 2
Supplementary Results .......... 4
Author Contributions .......... 15

**Abbreviations list**

BMP: bis(monoacylglycerol）phosphate
Cer: ceramides
FFA: free fatty acids
GM3: monosialogangliosides
LPA: lyso-PA
LPC: lyso-PC
LPE: lyso-PE
LPI: lyso-PI
LPS: lyso-PS
PA: phosphatidic acids
PC: phosphatidylcholines
PC-O: alkyl PC
PE: phosphatidylethanolamines
PE-O: alkyl PE
PG: phosphatidylglycerols
PI: phosphatidylinositols
PS: phosphatidylserines
SL: sulfatides
SM: sphingomyelins

**Table S1.** Amphiphilic compositions of scallop-derived plasmalogen-based LNP nanoformulations of vesicular (AN1) and hexosome (AN2) types. The natural scallop-derived plasmalogen extract (scPL70) has 70% vinyl ether phospholipid content and is characterized by the provider as a mixture of ethanolamine ether phospholipid (49.4%), choline ether phospholipid (24.9%), cholesterol (16.0%), and ceramide aminoethyl phosphonate (CAEP) (9.7%).

| Sample code | Amphiphilic compositions of lipid nanoparticles for *in vivo* studies* |
|---|---|
| **AN1** | scPL70 (0.2g)/Pluronic F127(0.04g) |
| **AN2** | scPL70 (0.2g)/Vitamin E (0.027g)/DOTAP (0.02g)/Pluronic F127(0.04g) |

*For *in vitro* cell culture experiments, the DOTAP lipid (promoting the adsorption of the nanoparticles in the nasal mucosa epithelium) was not included in the amphiphilic mixture. This small quantity did not modify the lipid nanoparticle structural organization for the *in vitro* tests.

**Supplementary Results**

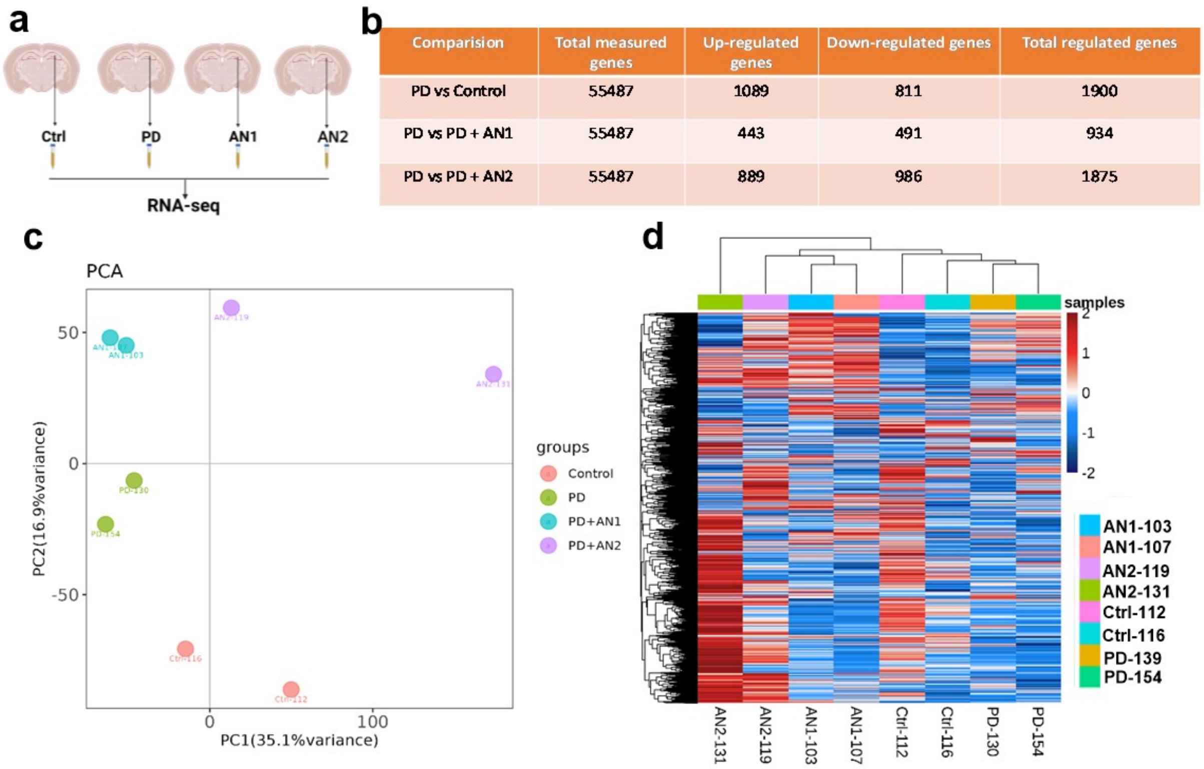


**Figure S1.** Transcriptomic analysis. **a**, Four groups of samples were prepared from hippocampus. The wild type was denoted as control, the PD mouse model as PD, the LNP AN1-treated group as (PD + AN1), and the AN2-treated group as (PD + AN2) (n=2). **b**, A summary statistics of RNA-seq results. **c**, Principal component analysis (PCA) of full transcriptomes. PC1/PC2 represents the % of determined variances. **d**, Expression heatmap based on the log2 transformation of the normalized count data of the whole set of genes.

The investigated two nanoformulations exhibited distinct effects on the transcriptomic results. The performed Gene Ontology Enrichment analysis revealed significant differences between AN1 and AN2 LNP-treated animals.

**Table S2.** TOP 10 of Gene Ontology Enrichment for PD *vs* (PD + AN1) and PD *vs* (PD+ AN2).

| **PD vs (PD + AN1)** | | **PD vs (PD + AN2)** | |
|---|---|---|---|
| **Terms** | **Adjusted P.Value** | **Terms** | **Adjusted P.Value** |
| cell chemotaxis | 0.00364344460530587 | neurotransmitter transport | 6.03983167119363e-12 |
| positive regulation of secretion by cell | 0.00364344460530587 | synapse organization | 7.10648148347602e-12 |
| mitochondrial ATP synthesis coupled electron transport | 0.00398530479629649 | vesicle-mediated transport in synapse | 7.10648148347602e-12 |
| aerobic electron transport chain | 0.00398530479629649 | regulation of neurotransmitter levels | 4.63136093571615e-11 |
| lipid transport | 0.00398530479629649 | synaptic vesicle cycle | 1.17506501885736e-09 |
| ATP synthesis coupled electron transport | 0.00398530479629649 | neurotransmitter secretion | 1.29314621007411e-09 |
| organic acid transport | 0.00398530479629649 | signal release from synapse | 1.29314621007411e-09 |
| electron transport chain | 0.00398530479629649 | dendrite development | 1.36674420353026e-09 |
| positive regulation of hormone secretion | 0.00470634902043119 | actin filament organization | 1.51323812984521e-09 |
| response to radiation | 0.00470634902043119 | synaptic vesicle exocytosis | 1.68617889050217e-09 |

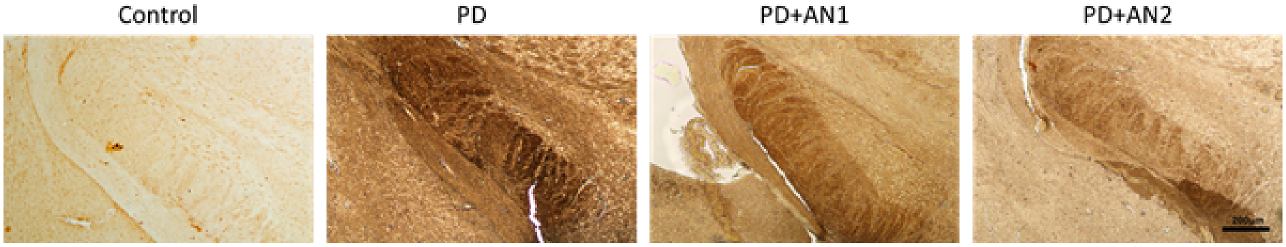


**Figure S2.** α-Synuclein staining in substantia nigra of the studied samples (Control, PD, PD+AN1, PD+AN2).

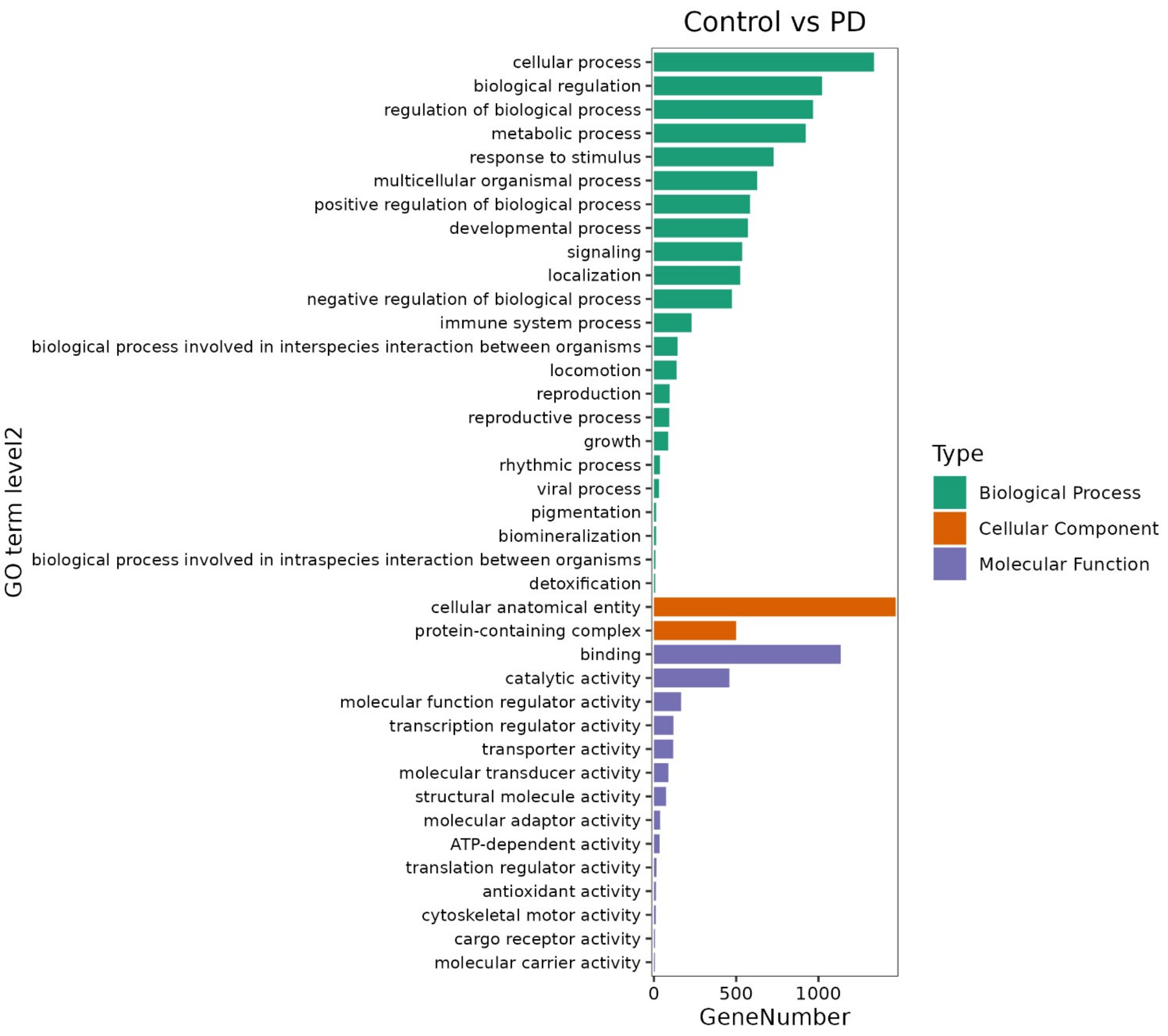


**Figure S3.** Gene Ontology (GO) enrichment analysis performed on differentially regulated genes with a comparison between the control and Parkinson's disease (PD) groups. The GO terms from the 'biological process,' 'cellular component', and 'molecular function' categories were listed and ranked.

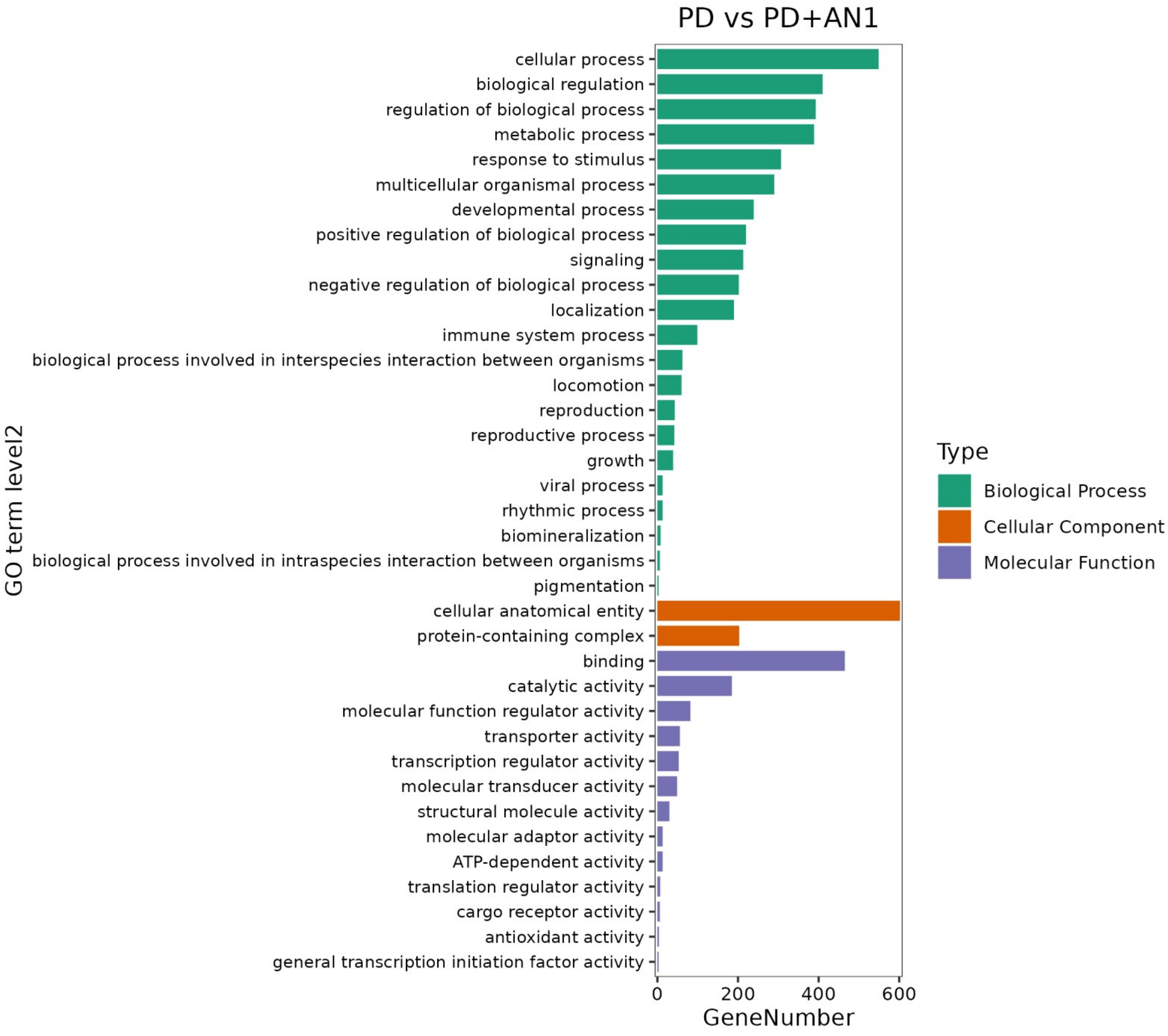


**Figure S4.** Gene Ontology (GO) enrichment analysis performed on differentially regulated genes with a comparison between the Parkinson's disease (PD) group and the LNP-treatment (PD + AN1) PD group. The GO terms from the 'biological process,' 'cellular component', and 'molecular function' categories were listed and ranked.

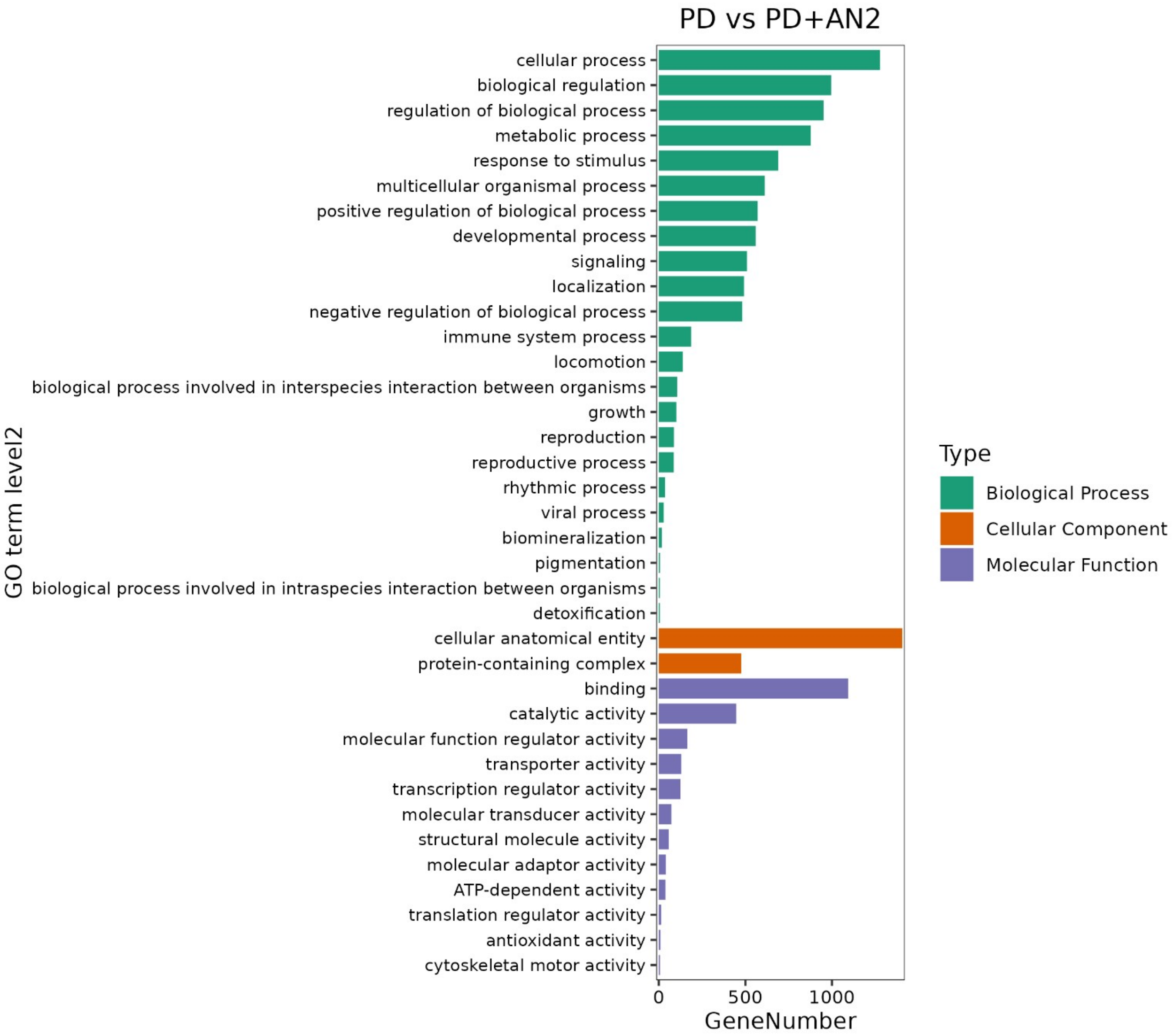


**Figure S5.** Gene Ontology (GO) enrichment analysis performed on differentially regulated genes with a comparison between the Parkinson's disease (PD) group and the LNP-treatment (PD) + AN2) PD group. The GO terms from the 'biological process,' 'cellular component', and 'molecular function' categories were listed and ranked.

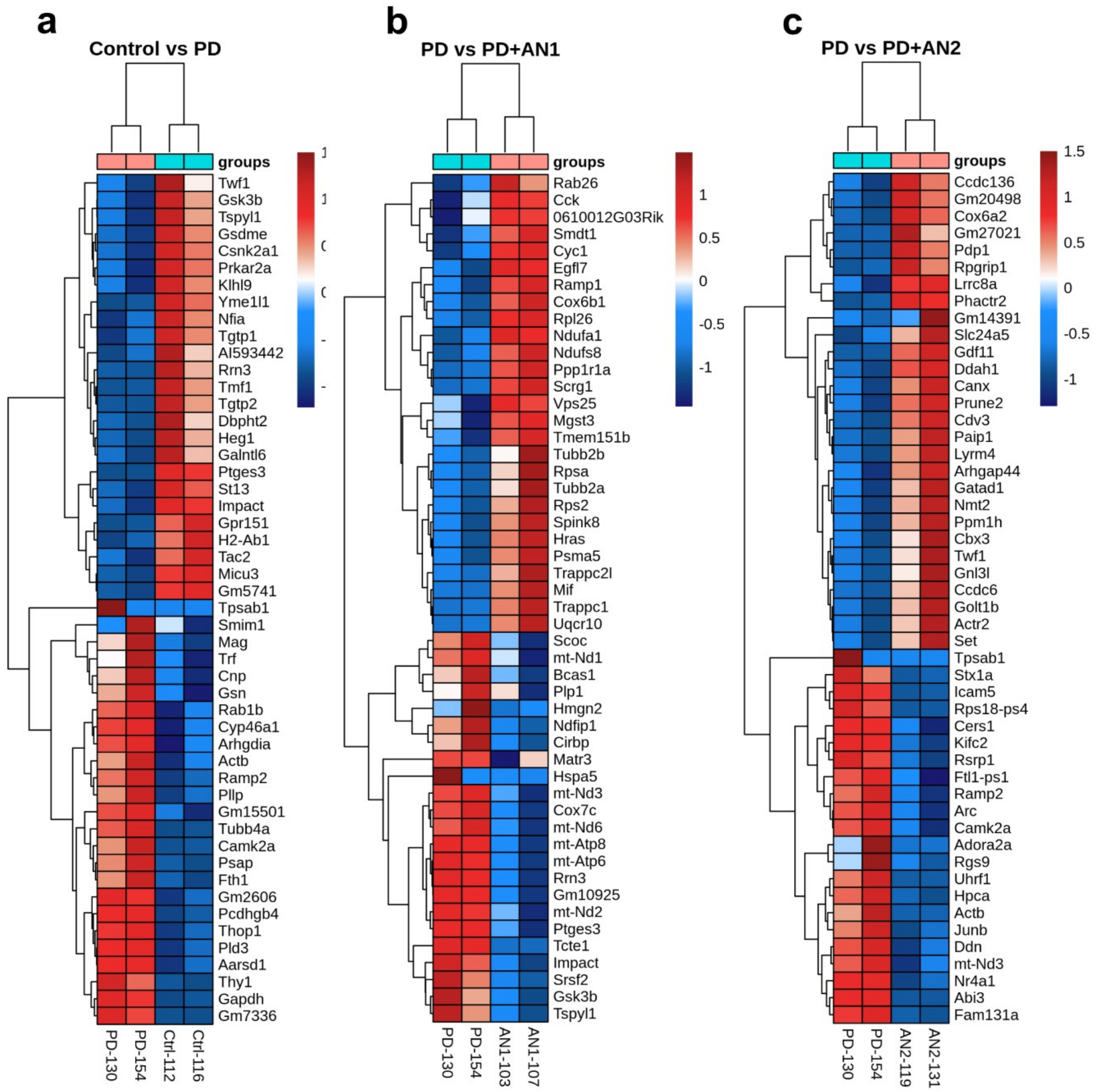


**Figure S6.** Heatmaps of top 50 differentially expressed genes with comparisons between **a**) control (WT) and PD, **b**) PD and (PD+AN1), and **c**) PD and (PD+ AN2) groups. The heatmaps show differentially expressed genes for the indicated every 2 groups. The color scale displays the fold change. Red indicates upregulation in gene expression, and blue indicates downregulation of gene expression.

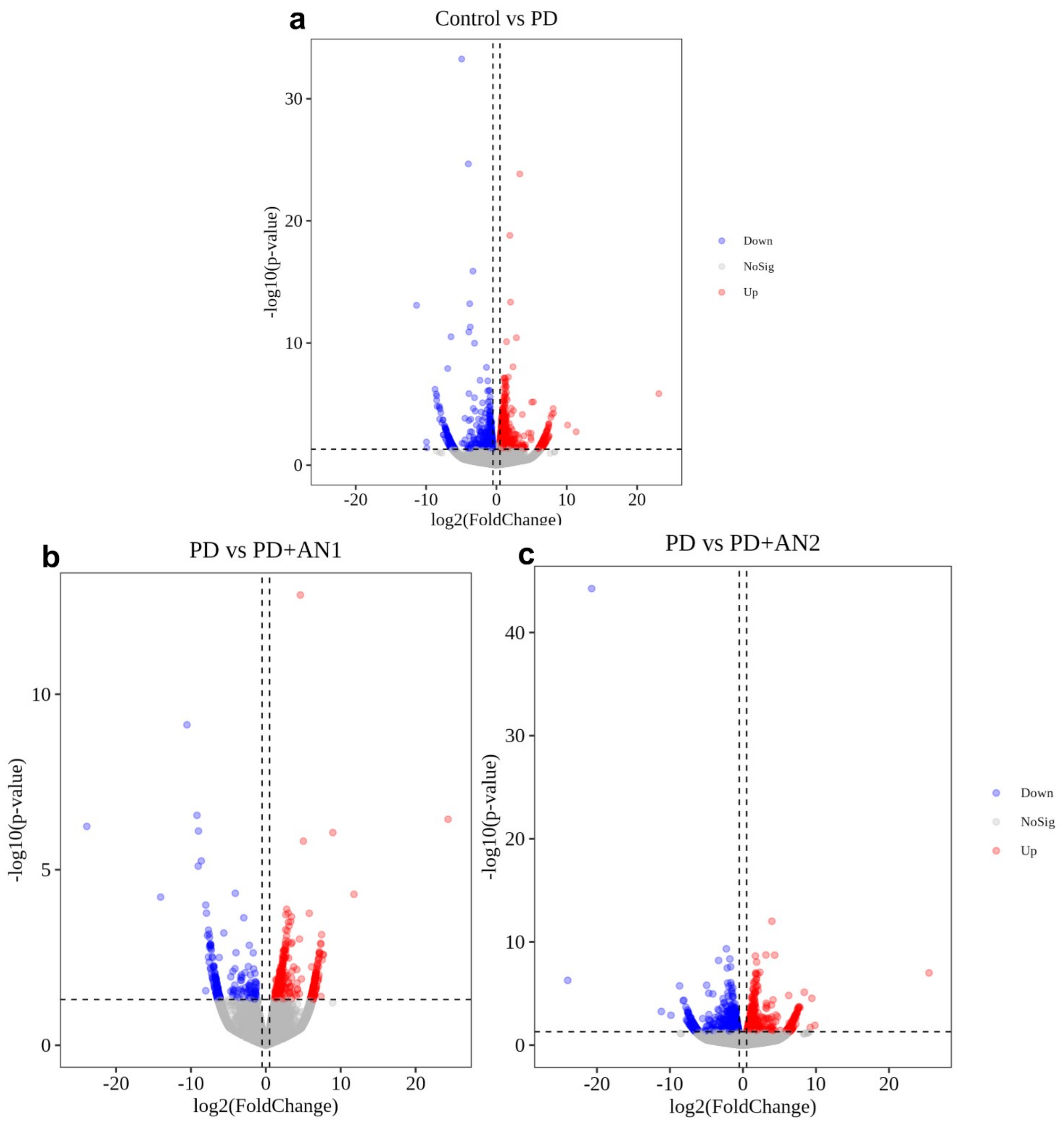


**Figure S7**. Volcano plots showing differentially expressed genes with comparisons between **a**) control and PD groups, **b**) PD and (PD+AN1) groups, and **c**) PD and (PD+AN2) groups. The X-axis represents the log2 fold change observed for each mRNA transcript and the Y-axis represents the log10 value of p-values of the significant test between replicates for each transcript. The color displays the fold change. Red indicates upregulation in gene expression, and blue indicates downregulation of gene expression. The grey color indicates no significant fold change.

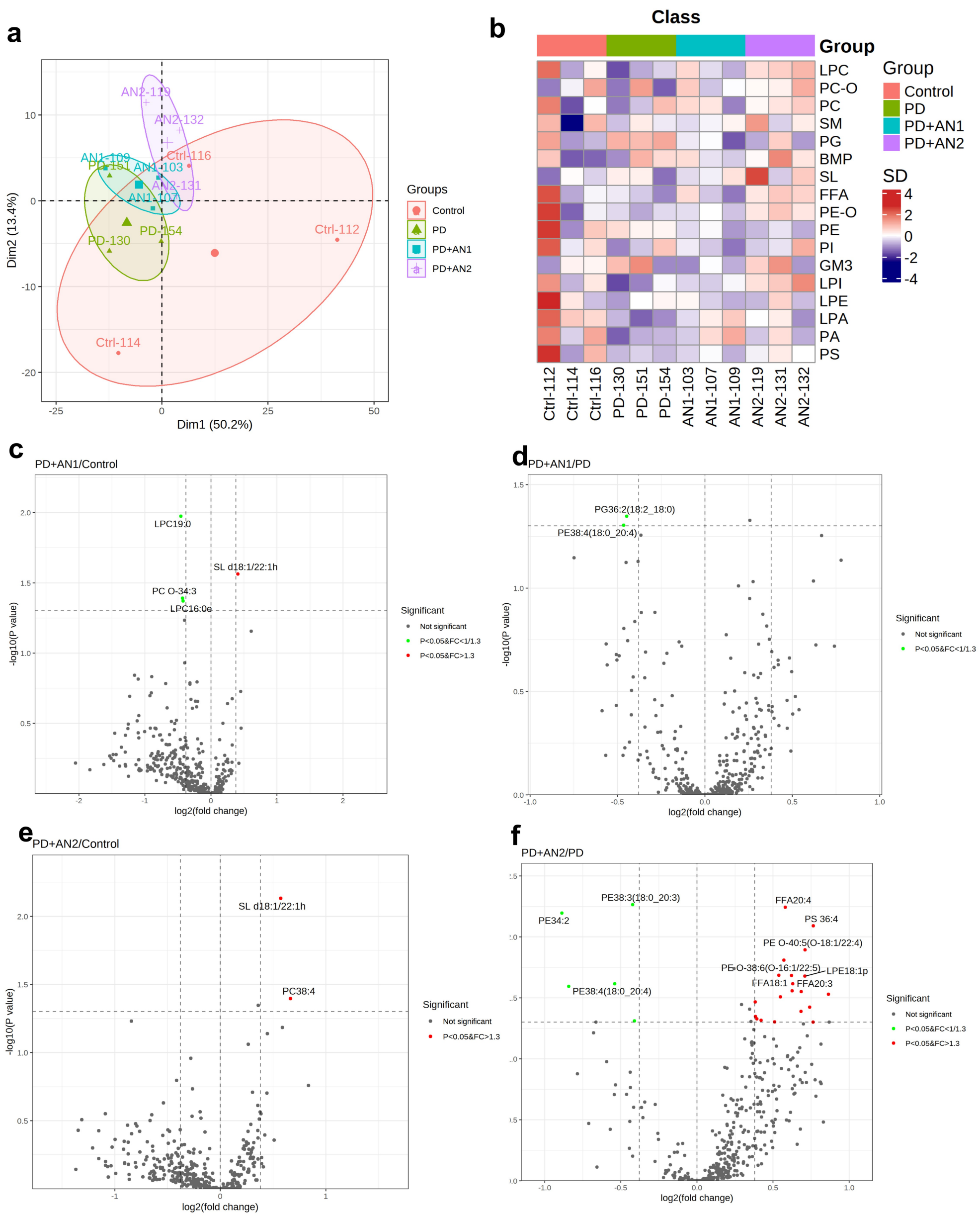


**Figure S8.** (**a**) Principal Component Analysis of Quality Control Samples Individual Plots based on lipidomic data. The percentages on the X/Y axes refer to the variance determined by that principal component. (**b**) Heat map of the levels of the different lipid classes in all samples. All the metabolites were standardized, resulting in the average value of each metabolite across all samples being close to 0 and appearing as white in the presented pattern. The intensity of the color indicates the degree of deviation from the average. Values below the average will be represented as negative, while values above the average will be positive, with darker colors indicating greater deviation from the average. The color bar on the right side of the heatmap represents the numerical values corresponding to colors, where the value's significance is linked to the standard deviation from the mean. (**c,d**) Volcano Plots of AN1 LNP-treated PD group as compared with PD mice and control groups, respectively. When metabolites meet both the p-value and fold change criteria (1.3), they will be marked in red if they are upregulated, and in green if they are downregulated. (**e,f**) Volcano Plots of AN2 LNP-treated PD group as compared with the PD mice and the control groups, respectively. When metabolites meet both the p-value and fold change criteria (1.3), they will be marked in red if they are upregulated, and in green if they are downregulated. The P-values were obtained using the Games-Howell test.

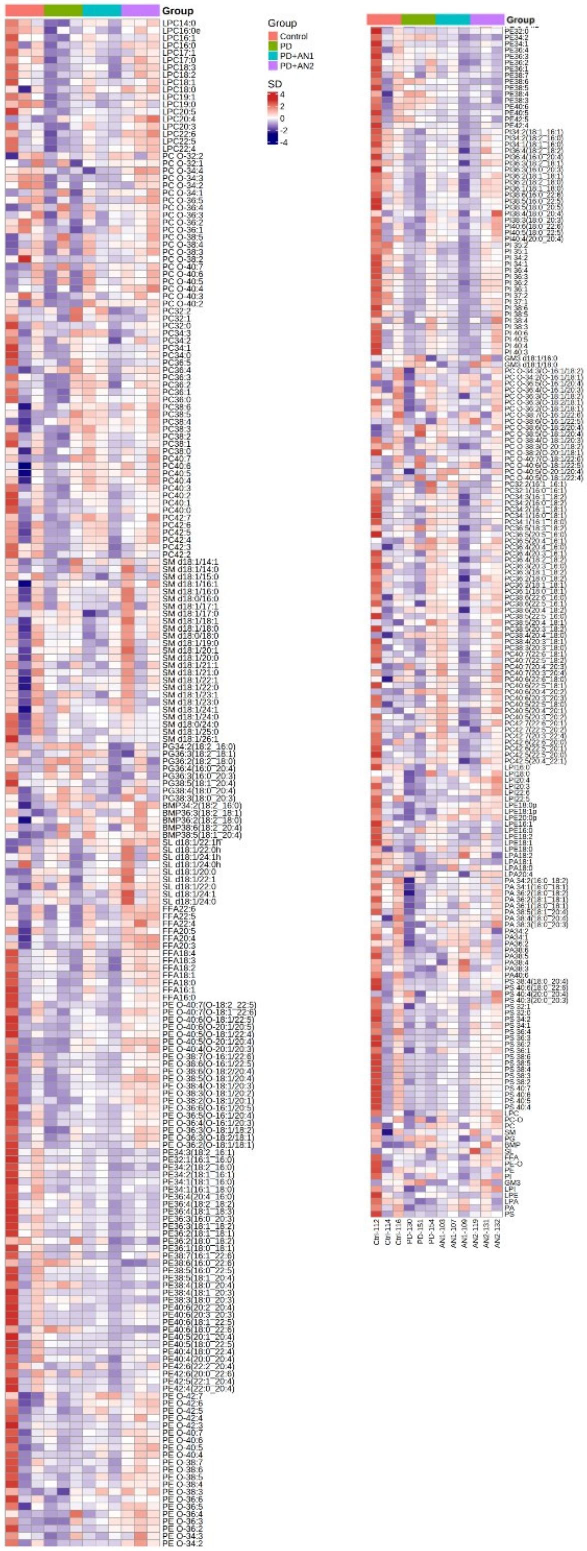


**Figure S9**. Heatmaps of altered lipid metabolites based on lipidomics analysis of control (WT), PD, (PD+AN1) and (PD+AN2) groups. The colors in the heat map indicated the log transformed values of each metabolites.

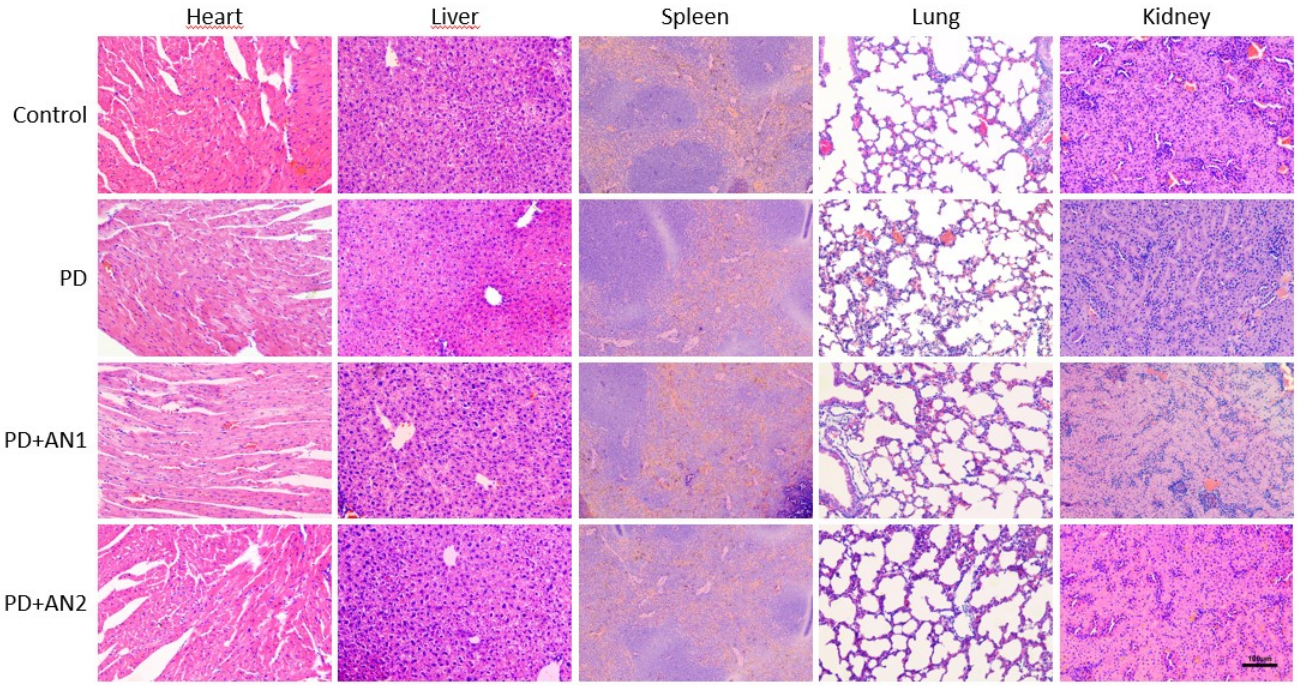


**Figure S10**. Histopathology of heart, liver, spleen, lung, and kidney organs for control (WT), PD, (PD+AN1), and (PD+AN2) groups.

## Author Contributions

A.A. and Y.D. conceived the project and designed the research. Y.W., A.A., and B.A. performed experiments and analyzed the data. J.W. assisted with the research. Y.D., B.A., and T.F. provided resources. Y.W., B.A., and A.A. designed the figures and interpreted the data. Y.W. prepared an initial draft. A.A., Y.W., M.S.H., and Y.D. wrote the paper. All authors contributed to the discussion of the results included in the article.
